# Selenium Nanoparticles as an Innovative Selenium Fertilizer Exert Less Disturbance to Soil Microorganisms

**DOI:** 10.3389/fmicb.2021.746046

**Published:** 2021-09-13

**Authors:** Jun Liu, Wen-Yu Qi, Hui Chen, Chao Song, Qiang Li, Shu-Guang Wang

**Affiliations:** ^1^Shandong Key Laboratory of Water Pollution Control and Resource Reuse, School of Environmental Science and Engineering, Shandong University, Qingdao, China; ^2^College of Agriculture and Forestry Science, Linyi University, Linyi, China; ^3^State Key Laboratory of Microbial Technology, Shandong University, Qingdao, China

**Keywords:** selenium nanoparticles, selenite, selenium fertilizer, soil microorganism, environmental persistent free radicals

## Abstract

Selenium (Se) is an essential trace element in the human body. Se-enriched agricultural products, obtained by applying Se fertilizer, are important sources of Se supplement. However, Se fertilizer may cause a series of environmental problems. This study investigated the transformation of exogenous selenium nanoparticles (SeNPs) and selenite (SeO_3_^2–^) in soil and explored their effects on soil microbial community and typical microorganisms. SeNPs exhibited a slow-release effect in soil, which promoted the growth of soil microorganisms and enriched soil probiotics. SeO_3_^2–^ was converted to a stable and low toxic state in soil, increasing persistent free radicals and decreasing microbial abundance and diversity. The influences of SeNPs and SeO_3_^2–^ on two typical soil microorganisms (*Bacillus* sp. and *Escherichia coli*) were also evaluated, and SeNPs were more difficult to enter into microorganisms directly, with lower toxicity and higher safety. These results indicated that SeNPs were a more environment-friendly Se additive for agriculture applications. This work provides useful information for better understanding the environmental fate and behavior of Se fertilizer in the soil.

## Introduction

Selenium (Se) is a micronutrient essential for human health (Rayman, 2000). The deficiency of Se can lead to cardiovascular disease, oxidative stress, decreased thyroid and immune functions, and an increased risk of various cancers (Zhou et al., 2018; Kumar and Prasad, 2021). Dietary uptake of Se from agricultural products is a major source of Se for humans (Evans et al., 2016). However, natural Se is insufficient in most areas of China, and billions of people are estimated to suffer Se deficiency (Terry et al., 2000; Tan J. et al., 2002). Therefore, Se-rich agricultural products are usually considered a feasible and effective way to improve the Se content in human bodies (Bhatia et al., 2013).

Se fertilizers, including inorganic [e.g., selenate, selenite (SeO_3_^2–^)] and organic (e.g., Se amino acid) varieties, have been widely used in soils to produce Se-rich crops (Alfthan et al., 2015). Generally, Se fertilizers effectively increase the Se content in plants (Kumar and Prasad, 2021). In addition, Se fertilizers could also promote the growth and development of plants, especially under stressful conditions such as high salinity and high temperature (Ashraf et al., 2018; Djanaguiraman et al., 2018). The early form of Se fertilizers was mainly SeO_3_^2–^. However, SeO_3_^2–^ is highly toxic in nature due to high bioavailability and high mobility (Rosenfeld et al., 2017). As new Se supplements, selenium nanoparticles (SeNPs), elemental form of Se at the nanoscale, have been demonstrated to be advantageous to the environment for their low toxicity and high efficiency (Skalickova et al., 2017). Owing to the application of exogenous Se fertilizers, the amount of Se in plow layer soil is increasing (Tan L.C. et al., 2016).

Soil microorganisms, especially rhizosphere microorganisms, are active in nutrient cycling and are key to the functioning of agricultural systems (Panke-Buisse et al., 2015). They play important roles in the degradation of organic matter, nitrogen fixation, and phosphate solubilization, all of which provide nutrients for plants (Martin et al., 2016; Valyi et al., 2016; Rosenblueth et al., 2018). In addition, soil microbes could facilitate plants to resist environmental stresses, such as drought (Guerena et al., 2019), extreme temperature (Liu et al., 2019), soil salinity (Ben Laouane et al., 2019), acidity (Tullio et al., 2019), alkalinity (Abd-Alla et al., 2014), and heavy metals (Benjelloun et al., 2019). Some microorganisms can also protect crops against diseases *via* producing antifungal metabolites (Weller et al., 2002). Moreover, soil microorganisms play an important role in the transformation and cycle of Se in nature. For example, some soil microorganisms (e.g., *Bacillus oryziterrae*, *Streptomyces* sp.) have been reported to metabolize and transform SeO_3_^2–^ to SeNPs (Bao et al., 2016; Tan Y. et al., 2016). Therefore, a stable and healthy soil microbial community is crucial for both plant growth and Se cycle. However, the effect of exogenous Se fertilizer on local soil microorganisms remains unknown. Therefore, it is necessary to explore the effect of Se fertilizer on soil microorganisms.

In this study, we investigated the transformation of exogenous SeNPs and SeO_3_^2–^ in soil and their effects on local soil microbial community and typical microorganisms. The transformation of Se in soil was determined by a continuous extraction method, and the changes in the abundance and diversity of soil microorganisms were analyzed using quantitative PCR (qPCR) and high-throughput sequencing. The core microbial population in the presence of exogenous Se was analyzed using linear discriminant analysis (LDA) effect size (LEfSe) and correlation network analysis. To further explore the influencing mechanism of SeNPs and SeO_3_^2–^ on the soil microbial community, persistent free radicals were detected using an electron paramagnetic resonance (EPR). In addition, the responses of two typical soil microorganisms (*Bacillus* sp. and *Escherichia coli*) to exogenous Se were investigated to further explore the biological effects of SeNPs and SeO_3_^2–^. This work completed a limited study on the transformation and effects of Se in soil, but the approach could be helpful for usage of SeNPs as a Se fertilizer for Se-rich agricultural products.

## Materials and Methods

### Reagents

Sodium SeO_3_^2–^, sodium hydroxide (NaOH), dipotassium phosphate (K_2_HPO_4_), monopotassium phosphate (KH_2_PO_4_), potassium persulfate (K_2_S_2_O_8_), sodium sulfite (Na_2_SO_3_), n-caprylic alcohol, acetic acid, and hydrochloric acid were obtained from Sinopharm (Shanghai, China). SeNPs were synthesized and purified using a recently described method (Shakibaie et al., 2010). In brief, the SeNPs were biologically reduced from SeO_3_^2–^ by microorganisms. The reduction product was washed and disrupted in liquid nitrogen. The suspension was then liquid–liquid extracted by n-caprylic alcohol. The purified SeNPs were collected from water phase. More details of synthesis purification and SEM observation were shown in Supplementary Text 1.

### Experimental Design

The soil used in this study was collected from a farmland in Jimo, Shandong province, and the physical and chemical properties of soil were shown in Supplementary Table 1. Five experiments with different Se treatment groups were established: (1) Control group, in which ddH_2_O was applied; (2) Low SeO_3_^2–^ group, in which 1 mmol/kg soil SeO_3_^2–^ solution was applied; (3) High SeO_3_^2–^ group, in which 10 mmol/kg soil SeO_3_^2–^ solution was applied; (4) Low SeNPs group, in which 1 mmol/kg soil SeNP suspension was applied; (5) High SeNPs group, in which 10 mmol/kg soil SeNP suspension was applied. The experiments were conducted in a greenhouse at 22°C, and the photoperiod was 16 h light and 8 h dark. Three soil samples were collected before all treatments were applied and designated as samples for Day 0. Soil samples of each treatment were collected in triplicate on Days 7 and 30 for further analysis.

### Determination of Available Forms of Selenium

The contents and existing forms of Se in soil samples were determined using a modified continuous extraction method (Martens and Suarez, 1997; Wright et al., 2003; Kulp and Pratt, 2004; Keskinen et al., 2009). Briefly, 1,000-g soil sample was placed in a 50-ml centrifuge tube and extracted using different solutions with a 1:10 solid/liquid ratio for each step. The water-soluble Se was extracted using H_2_O, and the ligand-exchangeable Se was extracted using 0.1 mol/L K_2_HPO_4_-KH_2_PO_4_ (pH 7.0). The alkali-soluble Se was extracted by 0.1 mol/L NaOH, and the elemental Se was extracted with the combination of 1 mol/L Na_2_SO_3_ (pH was adjusted to 7.0 by hydrochloric acid) and ultrasonication. The acid-soluble Se was extracted using acetic acid. The Se concentration in the extracts was detected with an atomic fluorescence photometer (AFS-933, Titan Instrument, Beijing, China). More details were described in Supplementary Text 2.

### DNA Extraction

Here, 0.25-g fresh soil sample was weighed to extract the total soil DNA. The extraction was performed using a TIANamp Soil DNA Kit (TIANGEN Biotech, Beijing, China). The DNA extracts were quantified and quality checked using a NanoDrop One UV–VIS Spectrophotometer (Thermo Fisher Scientific, United States). The DNA extracts were stored at −80°C for further analysis.

### Quantification of Total Bacteria in Soil and High-Throughput Sequencing

The abundance of total bacteria was assessed by quantification of 16S rRNA gene copies through qPCR. A universal primer set K90q/K94q (Supplementary Table 2) targeting bacterial 16S rRNA was employed. The protocol of qPCR followed a previously described method (Liu et al., 2020).

The V4–V5 region of the bacteria 16S rRNA gene was amplified with the primer set 515F/907R (details shown in Supplementary Table 2). In this step, an 8-bp-long barcode unique to each sample was appended. The PCR product was purified and quantified for the library generation. Then, the amplicon library was sequenced using an Illumina MiSeq platform at Shanghai Biozeron Biological Technology Co., Ltd. (Shanghai, China). Raw sequencing data were uploaded in NCBI Sequence Read Archive (SRA) database under accession number PRJNA664643.

### Bioinformatics and Statistical Analysis

High-throughput sequences were processed using the QIIME2 platform (version 2019.10) (Bolyen et al., 2019). Briefly, raw sequencing data were demultiplexed, filtered, and denoised. The denoised sequences were then clustered into operational taxonomic units (OTUs) with a 97% similarity. The representative sequences of each OTU were aligned against the SILVA database (SSU 135) with a confidence threshold of 70% to identify the taxonomy (Quast et al., 2013).

Alpha diversity indexes including Chao1, Shannon, and Simpson indices were calculated following the Vegan package in R. Principal coordinates analysis (PCoA) was calculated following the ape and GUniFrac package in R. LEfSe and correlation network analysis was conducted through the MicrobiomeAnalyst platform (Chong et al., 2020). LEfSe analysis was calculated using a significance level of *p* < 0.05 and an LDA score of 2. Correlation network analysis was performed at the genus level using the SparCC method with a significance level of *p* < 0.05 and a correlation threshold of 0.8 (Friedman and Alm, 2012; Jiang et al., 2017). According to the species classification data, the effects of Se on the function of soil microorganisms were predicted using the Tax4Fun2 algorithm.

### Measurements of Soil-Persistent Free Radicals

Fresh soil samples of 0.25 g were collected for EPR analysis. The samples were loaded into a quartz EPR tube, placed into the EPR resonator, and analyzed with a Bruker EMXnano spectrometer (Karlsruhe, Germany). The parameters were set as follows: center field, 3,420 G; sweep width, 80 G; sweep time, 40.06 s; receiver gain, 40 dB; modulation amplitude, 2.000 G; microwave frequency, 9.8 GHz; and microwave attenuation, 20.00 dB.

### Effect of Selenium Fertilizers on Typical Soil Microorganisms

Two typical soil microorganisms, namely, Gram-negative bacteria *Bacillus* sp. and Gram-positive bacteria *E. coli*, were chosen to investigate responses of different soil bacteria to exogenous Se. Briefly, the two bacteria were inoculated in a Luria-Bertani (LB) medium at a ratio of 1:100. SeNPs or SeO_3_^2–^ at a concentration of 0, 0.1, 0.5, 1, 5, and 10 mmol/L were added, respectively, and the mixed media were incubated at 37°C, 180 rpm for 24 h. OD_600_ was measured to characterize the biomass. Reactive oxygen species level was determined using Reactive Oxygen Species Assay Kit (Solarbio, Shanghai, China) following the manufacturer’s instructions. Glutathione peroxidase (GSH-Px) activity was determined using a Micro Glutathione Peroxidase Assay Kit according to the manufacturer’s protocol (Solarbio, Shanghai, China). Protein concentrations were determined following the bicinchoninic acid assay method (Smith et al., 1985).

## Results and Discussion

### Transformation of Selenium Nanoparticles and Selenite in Soil

**FIGURE 1:**
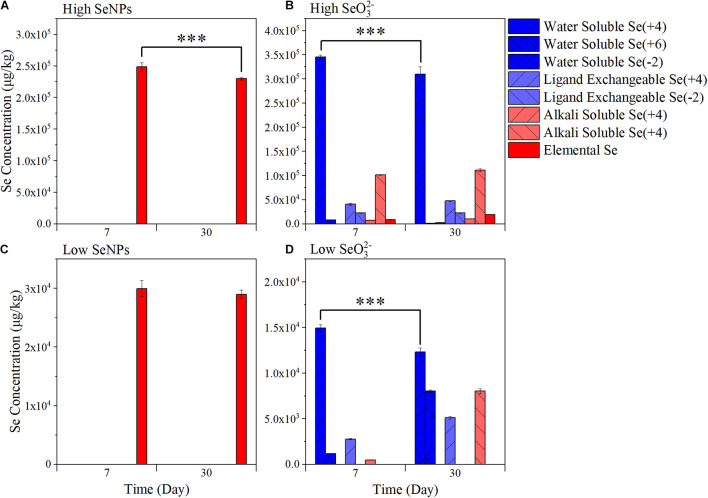
Variation of soil-available selenium (Se) fractions. **(A)** High selenium nanoparticles (SeNPs): the samples collected from the group treated with 10 mmol/kg soil SeNPs. **(B)** High selenite (SeO_3_^2–^): the samples collected from the group treated with 10 mmol/kg soil SeO_3_^2–^. **(C)** Low SeNPs: the samples collected from the group treated with 1 mmol/kg soil SeNPs. **(D)** Low SeO_3_^2–^: the samples collected from the group treated with 1 mmol/kg soil SeO_3_^2–^. Significant differences were analyzed by ANOVA; ^∗∗∗^*p* < 0.001.

The transformation of SeNPs and SeO_3_^2–^ in soil was analyzed with a continuous extraction method. As shown in Figure 1, the element Se decreased significantly along time in the High SeNP group from 248.58 mg/kg on Day 7 to 229.93 mg/kg on Day 30 (*p* < 0.001). In contrast, there was no significant difference in Se content in the Low SeNP group (29.98 mg/kg on Day 7, 28.99 mg/kg on Day 30). No soluble Se was detected in treatments with SeNPs. In both High SeO_3_^2–^ group and Low SeO_3_^2–^ group, the dominant form of Se was water-soluble SeO_3_^2–^, and its concentration decreased significantly along time (*p* < 0.001). Alkali-soluble Se (-II) was the second predominant Se form, which was reported to be mainly presented as organic-associated Se in soil (Keskinen et al., 2009). In addition, a relatively high elemental Se was observed in the High SeO_3_^2–^ group. Studies have shown that SeO_3_^2–^ is affected by redox potential in soil and can be reduced gradually by soil microorganisms, where the toxicity of Se decreases with decreasing valence (Piacenza et al., 2017; Wang et al., 2017; Zhai et al., 2021).

### Effects of Selenium Nanoparticles and Selenite on the Abundance and Diversity of Soil Microorganisms

**FIGURE 2:**
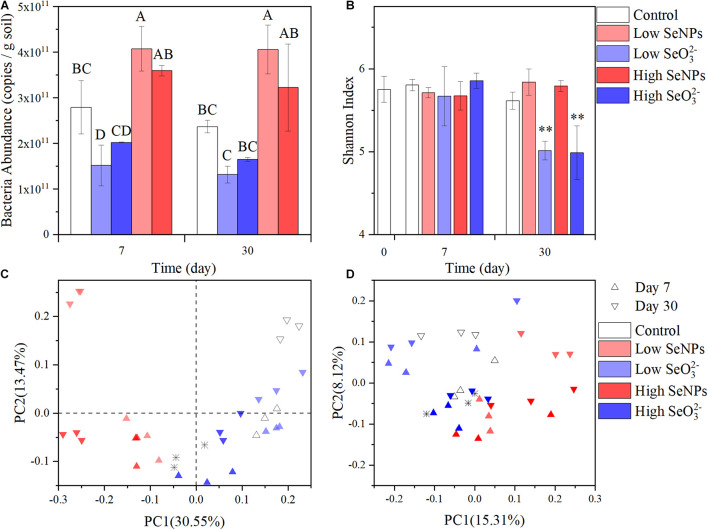
Abundance and diversity of the microbiota in each experimental group. **(A)** Total soil bacteria abundance. **(B)** Shannon Index. **(C)** Principal coordinates analysis (PCoA) plots of Bray–Curtis distances at the genus level. **(D)** PCoA plots of weighted UniFrac distances at the genus level. Control: the samples collected from the control group without selenium (Se) treatment. High selenite (SeO_3_^2–^): the samples collected from the group treated with 10 mmol/kg soil SeO_3_^2–^. Low SeO_3_^2–^: the samples collected from the group treated with 1 mmol/kg soil SeO_3_^2–^. High selenium nanoparticles (SeNPs): the samples collected from the group treated with 10 mmol/kg soil SeNPs. Low SeNPs: the samples collected from the group treated with 1 mmol/kg soil SeNPs. ^∗^ in **(C,D)** shows the data point of untreated soil. Significant differences were analyzed by ANOVA; ^∗∗^*p* < 0.01. Different letters indicate a significant difference (*p* < 0.05).

To explore the effect of SeNPs and SeO_3_^2–^ on the soil microbial community, soil samples were collected on Days 7 and 30. The abundance of soil bacteria was quantified by qPCR. The abundance of bacteria in the Control group remained relatively stable at 2.78–2.37 ^∗^ 10^11^ copies/g soil (Figure 2A). In the Low SeNP group, the abundance of soil bacteria increased by 146% (Day 7) and 171% (Day 30) compared with that of the Control group. A relatively smaller increase in the number of bacteria was observed in the High SeNP group, which was 129 and 136% on Days 7 and 30, respectively. However, the abundance of soil bacteria significantly decreased in treatments with SeO_3_^2–^, which was decreased by 45% (Day 7) and 55% (Day 30) in the Low SeO_3_^2–^ group. In the High SeO_3_^2–^ group, the higher concentration of SeO_3_^2–^ exhibited a stronger adverse effect on soil bacteria, where the abundance of bacteria was decreased by 70% on Day 30 compared with that of the Control group.

In this study, SeNPs promoted the development of soil microorganisms. It has been reported that SeNPs can play an antioxidant role in organisms by enhancing the activities of key enzymes in the antioxidant defense system (Zhai et al., 2017), activating antioxidant selenase (Guo et al., 2020), inhibiting protein glycosylation (Yu et al., 2015), or directly scavenging free radicals *in vivo*, therein promoting microbial growth. SeNPs are more stable in the environment and exert lower toxicity to microorganisms as a biological reduction product. However, the transformation of SeO_3_^2–^ in and out of microorganisms usually produces reactive oxygen species (Kessi and Hanselmann, 2004) that cause the toxicity of SeO_3_^2–^.

The effects of SeNPs and SeO_3_^2–^ on soil microbial community structure were further analyzed by 16S rRNA amplicon sequencing. A total of 1,488,614 sequences were obtained and assigned to 4,069 OTUs according to 97% similarity. No significant difference in α diversity was observed between the SeNP groups and the Control group (Figure 2B and Supplementary Table 3). According to PCoA of β diversity distance between samples (Figures 2C,D), the distance between the High/Low SeNP groups and the Control group was smaller than that between the SeO_3_^2–^ groups and the Control group. These results indicated that SeNPs exhibited no significant disturbance to the diversity of the soil microbial community.

However, there was a significant difference in the α diversity indexes between the High/Low SeO_3_^2–^ groups and the other groups (*p* < 0.01). The observed OTU number, Chao1 index, Simpson index, and Shannon index were all obviously reduced (Figure 2B and Supplementary Table 3). The High/Low SeO_3_^2–^ groups were separated from the other groups (Figures 2C,D). These results indicated that SeO_3_^2–^ affected the species and abundance of soil microorganisms. Further analysis of β diversity revealed a significant separation between the SeO_3_^2–^ groups and the Control group in the PCoA results based on the Bray–Curtis distance, which correlates with species abundance (Bray and Curtis, 1957; Figure 2C). However, PCoA results based on unweighted UniFrac distance, which is related to the developmental composition of species (Lozupone et al., 2011), showed no significant grouping differences, and the degree of interpretation among the principal components was low (Figure 2D). The different results of the two diversity distances implied that the SeO_3_^2–^ caused an abundance decrease but not extinction of soil microorganisms. It suggests that the effect of Se on soil microflora may not aim at specific species but be broad spectrum to all the soil microorganisms.

### Species Evolution and Soil Function Prediction in the Presence of Selenium Nanoparticles and Selenite

**FIGURE 3:**
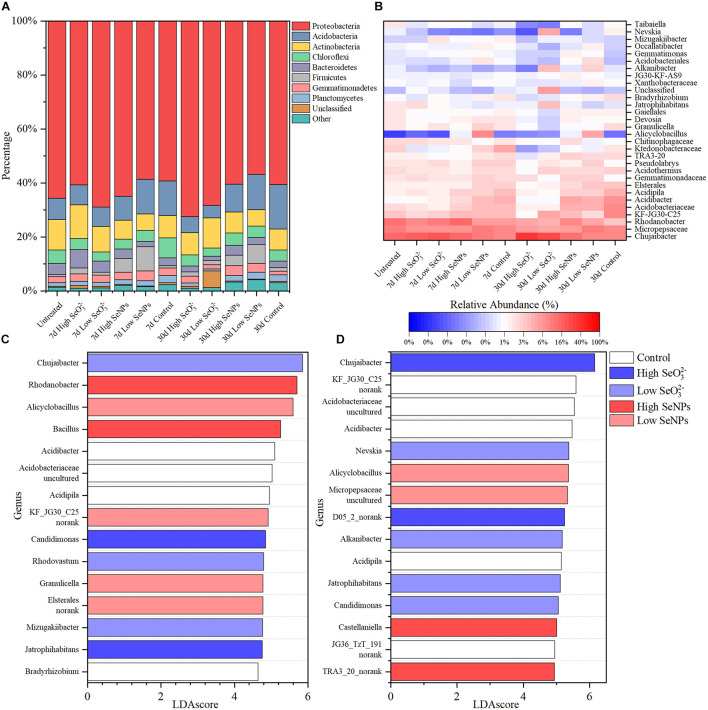
Taxonomy composition of the microbiota in each experimental group. **(A)** The relative abundance of major bacteria phyla. **(B)** The relative abundance of major bacteria genera. **(C,D)** The top 15 significantly changed genera identified by linear discriminant analysis effect size (LEfSe) on Days 7 **(C)** and 30 **(D)**. Control: the samples collected from the control group without selenium (Se) treatment. High selenite (SeO_3_^2–^): the samples collected from the group treated with 10 mmol/kg soil SeO_3_^2–^. Low SeO_3_^2–^: the samples collected from the group treated with 1 mmol/kg soil SeO_3_^2–^. High selenium nanoparticles (SeNPs): the samples collected from the group treated with 10 mmol/kg soil SeNPs. Low SeNPs: the samples collected from the group treated with 1 mmol/kg soil SeNPs.

As shown in Figure 3A, Proteobacteria dominated in soil samples from all treatment groups, with a relative abundance exceeding 50%. Significant differences were observed between the experimental groups at the genus level (Figure 3B). To quantitatively analyze the species changes, LEfSe method was used to analyze the species abundance at the genus level on Days 7 and 30 (Figures 3C,D), aiming to screen the species with significant differences in each experimental group. Under the given conditions, 205 and 267 significantly different species were identified, respectively. The relative abundance of several genera, e.g., *Acidibacter*, *Acidipila*, and *Bradyrhizobium*, was higher in the Control group, indicating that their relative abundance decreased under the stress of SeNPs and SeO_3_^2–^. The relative abundance of *Rhodanobacter*, *Bacillus*, *Alicyclobacillus*, and *Granulicella* increased in the SeNP groups, while the relative abundance of *Chujaibacter*, *Candidimonas*, *Jatrophihabitans*, and *Nevskia* increased in the SeO_3_^2–^ groups. The increased relative abundance may be due to the promotion effect of SeNPs or Se resistance of microorganisms.

**FIGURE 4:**
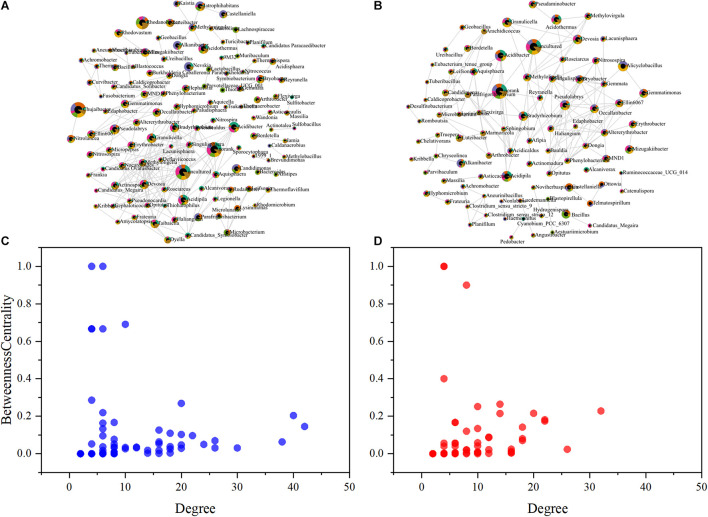
Co-occurrence network analysis showing the biological interactions at the family level under the treatment of selenite (SeO_3_^2–^) **(A)** and selenium nanoparticles (SeNPs) **(B)**. The size of the nodes is relative abundance. The scatter plot shows criteria of selecting for the keystone taxa in SeO_3_^2–^ **(C)** and SeNP **(D)** networks.

The influence of SeNPs and SeO_3_^2–^ on the soil microbial community was studied with a co-occurrence network method (Figure 4). Based on SparCC method, the correlation of soil microbial abundance was calculated, and then the graph inference of the network and the estimation of several topological characteristics were carried out. In the SeNP groups, the association network has 89 nodes and 308 connections, which can be classified into 16 constituent units (Figures 4A,C). In the SeO_3_^2–^ groups, there were 119 nodes, 442 connections, and 25 basic units (Figures 4B,D). To summarize, the impact of SeNPs on soil microbial network is less than that of SeO_3_^2–^. Based on intermediate centrality, the key microorganisms in each network were identified. In SeNP groups, *Tuberibacillus* and *Telmatospirillum* were the microorganisms located in the core of the network, while *Bryobacter* and *Mizugakibacter* were the microorganisms located in the core of the network in SeO_3_^2–^ groups.

The enriched microorganisms (e.g., *Bacillus*, *Alicyclobacillus*, *Granulicella*) in SeNP groups have been previously reported as soil probiotics (Zelenikhin et al., 2013; Long et al., 2018). In addition, *Tuberibacillus* and *Telmatospirillum* at the core of the microbial network are involved in the decomposition of soil organic matter and utilization of carbon, nitrogen, phosphorus, and other elements (Hatayama et al., 2006; Xue et al., 2017; Ding et al., 2019), indicating that the addition of SeNPs induces active probiotics related to basic soil functions and improves the soil microbial community. *Chujaibacter*, *Candidimonas*, *Jatrophihabitans*, *Nevskia*, *Bryobacter*, and *Mizugakiibacter*, which were enriched in the SeO_3_^2–^ groups or lived in the core of the network, have the ability to treat heavy metals and resist UV, antibiotics, and other biological or abiotic stresses (Vaz-Moreira et al., 2011; Větrovský and Baldrian, 2015; Prakash et al., 2020; Sánchez-Soto et al., 2020). These results imply that SeO_3_^2–^ appeared to be toxic to the soil microbial community.

The effects of exogenous Se on soil functions were predicted based on the Tax4Fun2 algorithm (Supplementary Figure 2). There was no significant difference between treatments, indicating that exogenous Se exhibited little impact on soil function. Therefore, the soil microbial community can effectively resist the external interface and maintain the ecological functions of the soil, reflecting the robustness of the soil microbial community function.

### Persistent Free Radicals Release From Soil Induced by Selenium Nanoparticles and Selenite

**FIGURE 5:**
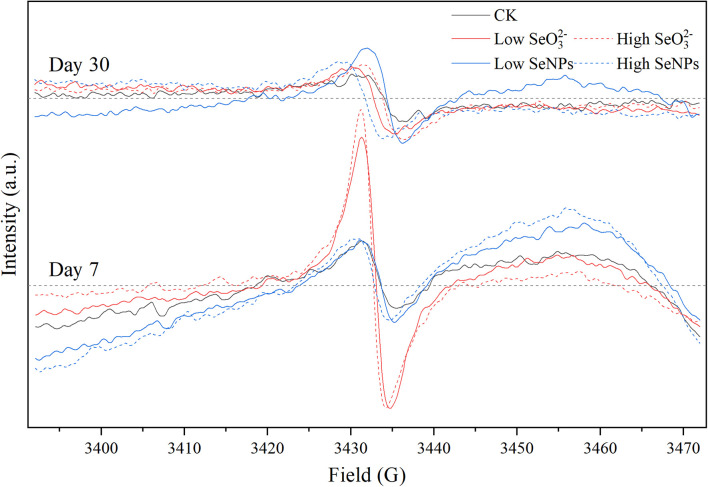
Electron paramagnetic resonance (EPR) spectra of each experimental group on Days 7 and 30.

In this study, exogenous SeNPs and SeO_3_^2–^ could change soil microbial community obviously. To explore the influencing mechanism of SeNPs and SeO_3_^2–^ on the soil microbial community, persistent free radicals in the soil were determined, as the migration and transformation of Se may induce the release of free radicals (Kessi and Hanselmann, 2004). By analyzing the G-factor of free radical signal, it was found that the persistent free radicals in soil were mainly oxygen-center free radicals and semi-quinone free radicals (Khachatryan and Dellinger, 2011; Figure 5 and Supplementary Table 4). There was no significant difference in free radical signals between the SeNP groups and the Control group. However, in the SeO_3_^2–^ groups, the persistent free radical signal in the soil was significantly enhanced on Day 7, but there was no significant difference on Day 30. Nevertheless, the bacterial community showed a more significant difference on Day 30, indicating that the corresponding response of the soil microbial community to exogenous Se compounds lagged behind the migration and transformation of exogenous Se in soil. The migration and transformation of exogenous Se in the environment, especially SeO_3_^2–^, is mainly due to chemical reactions, but the changes of soil microbial community need a longer period compared with chemical reactions.

### Effects of Selenium Nanoparticles and Selenite on Typical Soil Microorganisms

**FIGURE 6:**
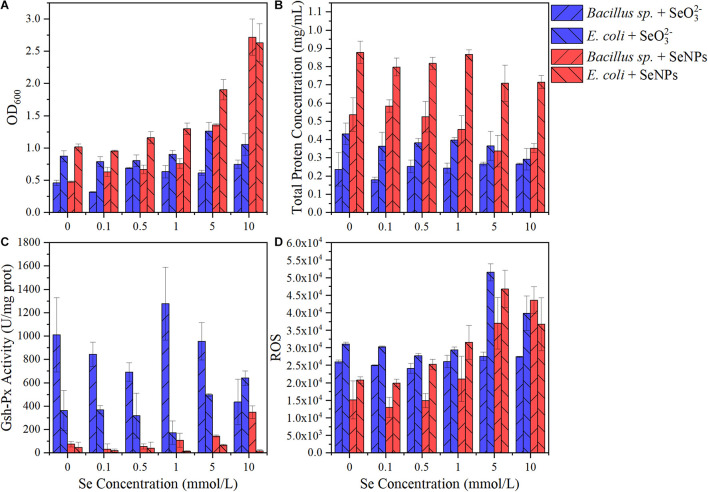
Effects of selenium nanoparticles (SeNPs) and selenite (SeO_3_^2–^) on typical soil microorganisms. **(A)** OD_600_. **(B)** Total protein concentration. **(C)** Glutathione peroxidase (GSH-Px) activity. **(D)** Reactive oxygen species.

In this study, the application of SeNPs and SeO_3_^2–^ exhibited different effects on the soil microbial community. SeNPs promoted the propagation of soil probiotics, while SeO_3_^2–^ induced the enrichment of microorganisms with a higher tolerance to environmental stress. In addition, we found that the relative abundance of Gram-negative bacteria increased more than that of Gram-positive bacteria after SeNP and SeO_3_^2–^ treatments. Therefore, we selected two representative soil microorganisms (*Bacillus* sp. and *E. coli*) to investigate their response to exogenous Se. As shown in Figure 6A, the microbial biomass increased with the increase of SeNP concentration, while SeO_3_^2–^ at different concentrations had little influence on the microbial biomass. Regardless of the Se concentration, both tested bacteria showed higher growth in treatments with SeNPs compared with that of SeO_3_^2–^ groups. In addition, experimental groups with *E. coli* always showed higher biomass than that with *Bacillus* sp. Similar to the results of biomass, the total protein concentration in SeNP treatment groups was higher than that of SeO_3_^2–^ treatment group, and the total protein content of *E. coli* was higher than that of *Bacillus* sp. (Figure 6B). The above results indicated that SeNPs promoted the growth of microorganisms at a certain concentration compared with SeO_3_^2–^, and the promotion was more significant in *E. coli*. We further determined the intracellular reactive oxygen species of different treatment groups and found that the intracellular reactive oxygen species of *Bacillus* sp. were lower than that of *E. coli* (Figure 6D). It implies that *E. coli* had a higher ability to resist oxidative stress, which is consistent with its higher biomass.

GSH-Px is a kind of antioxidant enzyme with Se atom as its active center, and its activity can characterize the bioavailability of Se (Yu and Wei, 2013). The GSH-Px activity in the SeNP groups was significantly lower than that in the SeO_3_^2–^ treatment groups (Figure 6C). Although GSH-Px activity was significantly different (*p* < 0.05), there was no significant correlation between enzyme activity and Se concentration (Figure 6C and Supplementary Figure 3). The GSH-Px activity of *Bacillus* sp. was higher than that of *E. coli*, partly resulting in the lower amount of intracellular reactive oxygen species in *Bacillus* sp.

The results indicated that SeNPs were less bioavailable than SeO_3_^2–^. It has been reported that SeO_3_^2–^ can be easily transported across the membrane *via* binding to the sulfhydryl sites on the cell membrane or by phosphate transfer protein (Yu et al., 2018; Zhu et al., 2020). In contrast, SeNPs are in a reduced state. The Se atom is coated with a layer of extracellular polymer (Avendaño et al., 2016), making it difficult to enter the microorganism directly. It may partly explain the lower toxicity of SeNPs.

## Conclusion

In this study, the migration and transformation of two Se fertilizers (i.e., SeNPs and SeO_3_^2–^) in soil were determined. Their effects on the soil microbial community were assessed, and the possible mechanism was analyzed. It was found that SeNPs were more stable in the soil, while SeO_3_^2–^ would be gradually transformed into a more stable and lower toxicity state in the soil. SeNPs promoted the propagation of soil probiotics, while SeO_3_^2–^ reduced the abundance and diversity of microorganisms possibly due to the toxicity of the persistent free radicals released during the transformation of SeO_3_^2–^. Experiments with two typical soil microorganisms showed that SeNPs showed lower toxicity and higher safety because SeNPs enter microorganisms slowly. These results indicated that SeNPs were a more environment-friendly Se additive for agricultural application. This work provides useful information for better understanding of the environmental fate and behavior of Se fertilizer in the soil.

## Data Availability Statement

The datasets presented in this study can be found in online repositories. The names of the repository/repositories and accession number(s) can be found below: https://www.ncbi.nlm.nih.gov/bioproject/ PRJNA664643.

## Author Contributions

JL and QL conceptualized and designed the study. JL and W-YQ carried out the experimental work and described in the article. JL analyzed, interpreted, and visualized the data. JL, CS, and HC drafted the manuscript. S-GW acquired the funding. All authors contributed to revise the manuscript and approved the submitted version, with proofreading performed by JL.

## Conflict of Interest

The authors declare that the research was conducted in the absence of any commercial or financial relationships that could be construed as a potential conflict of interest.

## Publisher’s Note

All claims expressed in this article are solely those of the authors and do not necessarily represent those of their affiliated organizations, or those of the publisher, the editors and the reviewers. Any product that may be evaluated in this article, or claim that may be made by its manufacturer, is not guaranteed or endorsed by the publisher.
