# Supplementary material for: Selenium Nanoparticles as an Innovative Selenium Fertilizer Exert Less Disturbance to Soil Microorganisms
Source: Front Microbiol. 2021 Sep 13;12:746046. doi: 10.3389/fmicb.2021.746046 (PMC8473918; doi:10.3389/fmicb.2021.746046)
Supplement: Supplementary file 1 [file Presentation_1.pdf]

Supplementary Material

List of supplementary texts, figures and tables:

Supplementary Text 1 Biosynthesis isolation recovery and SEM observations of SeNPs

Supplementary Text 2 Continuous selenium extraction method

Supplementary Figure 1 SEM images and corresponding EDS spectra of bacteria culture with SeNPs (a) and the purified SeNPs (b)

Supplementary Figure 2 Functional community composition by each experimental group

Supplementary Figure 3 Correlation analysis among bacterial species, Se species, Se concentration and Bacteria physiological indexes. *. **.***. represent significant correlation at the level of 0.05, 0.01 and 0.001, respectively.

Supplementary Table 1 Physical and chemical properties of experimental soil

Supplementary Table 2. Primers used for PCR amplifications in this study

Supplementary Table 3 Alpha diversity indexes of each experimental group

Supplementary Table 4 g-factor of EPR

Supplementary Text 1 Biosynthesis isolation recovery and SEM observations of SeNPs

Synthesis of Se NPs was carried out by Bacillus subtilis 168 using a recently described method. The sterilized LB broth was supplemented with Sodium selenite (500 mmol L^{-1}, which is sterilized by a filtration process using a 0.22 μm Millipore filter apparatus). The medium was inoculated (1% v/v) with the fresh inoculums (OD600, 0.1) and for aerobic cultivation; the flasks were plugged with cotton and incubated at 30°C in a shaker incubator (150 rpm). After 14 h, the bacterial cells and SeNPs were removed from the culture medium by centrifugation at 4000g for 10 min.

The resulting pellets were washed with 0.9% NaCl solution, centrifuged and transferred to a mortar. By adding liquid nitrogen, the pellets were frozen and disrupted with a pestle. The resulting slurry was ultrasonicated at 100 W for 5 min and washed three times by sequential centrifugation (10000g, 5 min) using Tris-HCl buffer (1.5 M, pH 8.3) containing 1% sodium dodecyl sulfate (SDS) and deionized water, respectively. The pellets were suspended in deionized water, and the resulting suspension containing SeNPs, and cell debris was collected. The suspension (4 mL) was then transferred to test tubes, and 2 mL of n-octyl alcohol was added to each tube. The assortments were shaken vigorously, the mixed phases were separated by centrifugation at 2000g for 5 min and were stored at 4°C for 24 h. Following a period, the generated SeNPs can be observed at the bottom of the tubes and the cell debris remained between two phases. The lower and upper phases were discarded, and settled SeNPs were washed with chloroform, ethyl alcohol and distilled water, respectively. For further characterizations and biological experiments, the cleaned SeNPs were then resuspended in deionized water and stored at 4°C.

The bacterial cells with SeNPs were collected by centrifugation and washed three times using PBS. The washed pellets were fixed overnight in a solution of 2.5% glutaraldehyde in PBS. The fixed samples were dehydrated using elevated contents of ethanol in distilled water (30%, 50%, 75%, 90%, and 100%). The dehydrated bacterial cells and the cleaned SeNPs were dropped on a copper sheet and then air-dried. The prepared samples were further characterized by SEM-EDX (Quanta 250 FEG; FEI, Hillsboro, USA) in secondary electron mode at 20 kV accelerating voltage with a working distance of 4–5 mm. The SEM image was shown in Fig. S1.

Supplementary Text 2 Continuous selenium extraction method

This method is based on the research of Martens, Wright, Keskinen, Kulp et al (Martens and Suarez, 1997; Wright et al., 2003; Kulp and Pratt, 2004; Keskinen et al., 2009). The method was based on the difference of the binding degree of different forms of selenium in the soil. A variety of extracts were used to extract the tested samples continuously, and the selenium content was detected by atomic fluorescence photometer. The detailed method is as follows:

Water Soluble Se

Most of Se (VI), some of Se (IV) and a small amount of water-soluble organic selenium can be extracted with water as extraction agent. A 1.0g soil sample was weighed into a 50 mL centrifuge tube, 10 mL H_2O was added and shaken for 2 h. The sample suspension was centrifuged at 10000g for 10 min, and the precipitation (1) was separated from the supernatant for later use. The obtained precipitate was washed by adding 10 mL H_2O, resuspended, and centrifuged. The supernatant obtained by centrifugation was merged into supernatant (I), and H_2O was added to 25 mL at constant volume. 0.05 mL supernatant (1) was added with 5% hydrochloric acid (current-carrying concentration) to 10 mL, and atomic fluorescence quantification was completed within 20 min. 0.05 mL of Se (VI) supernatant (1) was taken, and 1 mL of 6 mol/L hydrochloric acid was added. The solution was heated in a water bath at 90 ° C for 30 min to reduce Se (VI) to Se (IV), and H_2O was added to 10 mL at constant volume. The concentration was measured by machine. 0.05 mL supernatant (1) was taken, 0.5ml 0.1 mol/L $K_2S_2O_8$ was added and heated in a water bath at 90 °C for 30 min to oxidize all Se into Se (VI). Then 1 mL concentrated hydrochloric acid was added and heated in a water bath at 90 °C for 30 min. Reduce Se (VI) to Se (IV), add H_2O to 10 mL at constant volume, and measure the concentration on the machine.

Ligand Exchangeable Se

Se (IV), which is closely bound with iron and manganese oxides and clay minerals, can be extracted by using phosphate buffer as the extractant. 10 mL 0.1 mol/L K_2HPO_4-KH_2PO_4 (pH 7.0) was added to the precipitation (1) for 2 h. The sample suspension was centrifuged at 10000g for 10 min, and the precipitation (2) was separated from the supernatant for later use. The obtained precipitate was washed by adding 10 mL H_2O, resuspended, and centrifuged. The supernatant obtained by centrifugation was merged into supernatant (2), and H_2O was added to 25 mL at constant volume. 0.05 mL of supernatant (2) was taken, and 5% hydrochloric acid (current-carrying concentration) was added to 10 mL. Atomic fluorescence quantification was completed within 20 min. 0.05 mL supernatant (2) was taken, 0.5 mL 0.1 mol/L $K_2S_2O_8$ was added and heated in a water bath at 90 °C for 30 min to oxidize all Se into Se (VI). Then 1mL concentrated hydrochloric acid was added and heated in a water bath at 90 °C for 30 min. Reduce Se (VI) to Se (IV), add H_2O to 10 mL at constant volume, and measure the concentration on the machine.

Alkali soluble selenium/organic selenium

0.1 mol/L NaOH can effectively extract alkali-soluble organic selenides, adsorbed organic selenium and complexed organic selenium from soil. Add 10 mL 0.1mol/L NaOH to the precipitate (2) and heat it in a water bath at 90 ℃ for 2 h. The sample suspension was centrifuged at 10000g for 10 min, and the precipitation (3) was separated with the supernatant for later use. The obtained precipitate was washed by adding 10 mL H_2O, resuspended, and centrifuged. The supernatant obtained by centrifugation was merged into supernatant (3), and H_2O was added to 25 mL at constant volume. 0.05 mL supernatant (3) was added with 5% hydrochloric acid (current-carrying concentration) to 10 mL, and atomic fluorescence quantification was completed within 20min. 0.05 mL of supernatant (3) was taken, and 1 mL of 0.1 mol/L $K_2S_2O_8$ was added and heated in a water bath at 90 °C for 30 min to oxidize all Se into Se (VI). Then 1 mL of 6 mol/L hydrochloric acid was added and heated in a water bath at 90 ° C for 30 min. Reduce Se (VI) to Se (IV), add H_2O to 10 mL at constant volume, and measure the concentration on the machine.

Elemental selenium

The combination of 1 mol/L Na_2SO_3 and ultrasonication can effectively dissolve elemental selenium but has no significant effect on organic selenium and selenide minerals. Add 10 mL 1 mol/L Na_2SO_3 (hydrochloric acid adjusted to pH 7.0) to the precipitate (3) and ultrasonication for 8 h. The sample suspension was centrifuged at 10000 g for 10 min, and the precipitation (4) was separated with the supernatant for later use. The obtained precipitate was washed by adding 10 mL H_2O, resuspended, and centrifuged. The supernatant obtained by centrifugation was merged into supernatant (4), and H_2O was added to 25 mL at constant volume. 0.05 mL of supernatant (4) was taken and 1 mL of 0.1 mol/L $K_2S_2O_8$ was added and heated in a water bath at 90 ℃ for 30 min to oxidize all Se into Se (VI). Then 1 mL of 6 mol/L hydrochloric acid was added and heated in a water bath at 90 ℃ for 30 min. Reduce Se (VI) to Se (IV), add H_2O to 10 mL at constant volume, and measure the concentration on the machine.

Selenium in acid solution

The use of acetic acid can destroy carbonate and iron and manganese oxides and dissolve the bound selenium in them. 15% acetic acid was added to the precipitate (4) for 2 h. The sample suspension was centrifuged at 10000 g for 10 min, and the precipitation (5) was separated with the supernatant for later use. The obtained precipitate was washed by adding 10 mL H_2O, resuspended, and centrifuged. The supernatant obtained by centrifugation was merged into supernatant (5), and H_2O was added to 25 mL at constant volume. 0.05 mL of supernatant (5) was taken, and 5% hydrochloric acid (current-carrying concentration) was added to 10 mL. Atomic fluorescence quantification was completed within 20min. 0.05 mL of supernatant (5) was taken and 1 mL of 0.1 mol/L $K_2S_2O_8$ was added and heated in a water bath at

90 ℃ for 30 min to oxidize all Se into Se (VI). Then 1 mL of 6 mol/L hydrochloric acid was added and heated in a water bath at 90 ℃ for 30 min. Reduce Se (VI) to Se (IV), add H_2O to 10 mL at constant volume, and measure the concentration on the machine.

Reference

Keskinen, R., Ekholm, P., Yli-Halla, M., and Hartikainen, H. (2009). Efficiency of different methods in extracting selenium from agricultural soils of Finland. *Geoderma* 153, 87–93. doi:10.1016/j.geoderma.2009.07.014.

Kulp, T. R., and Pratt, L. M. (2004). Speciation and weathering of selenium in upper cretaceous chalk and shale from South Dakota and Wyoming, USA. *Geochim. Cosmochim. Acta* 68, 3687–3701. doi:10.1016/j.gca.2004.03.008.

Martens, D. A., and Suarez, D. L. (1997). Selenium speciation of soil/sediment determined with sequential extractions and hydride generation atomic absorption spectrophotometry. *Environ. Sci. Technol.* 31, 133–139. doi:10.1021/es960214+.

Wright, M. T., Parker, D. R., and Amrhein, C. (2003). Critical evaluation of the ability of sequential extraction procedures to quantify discrete forms of selenium in sediments and soils. *Environ. Sci. Technol.* 37, 4709–4716. doi:10.1021/es0342650.

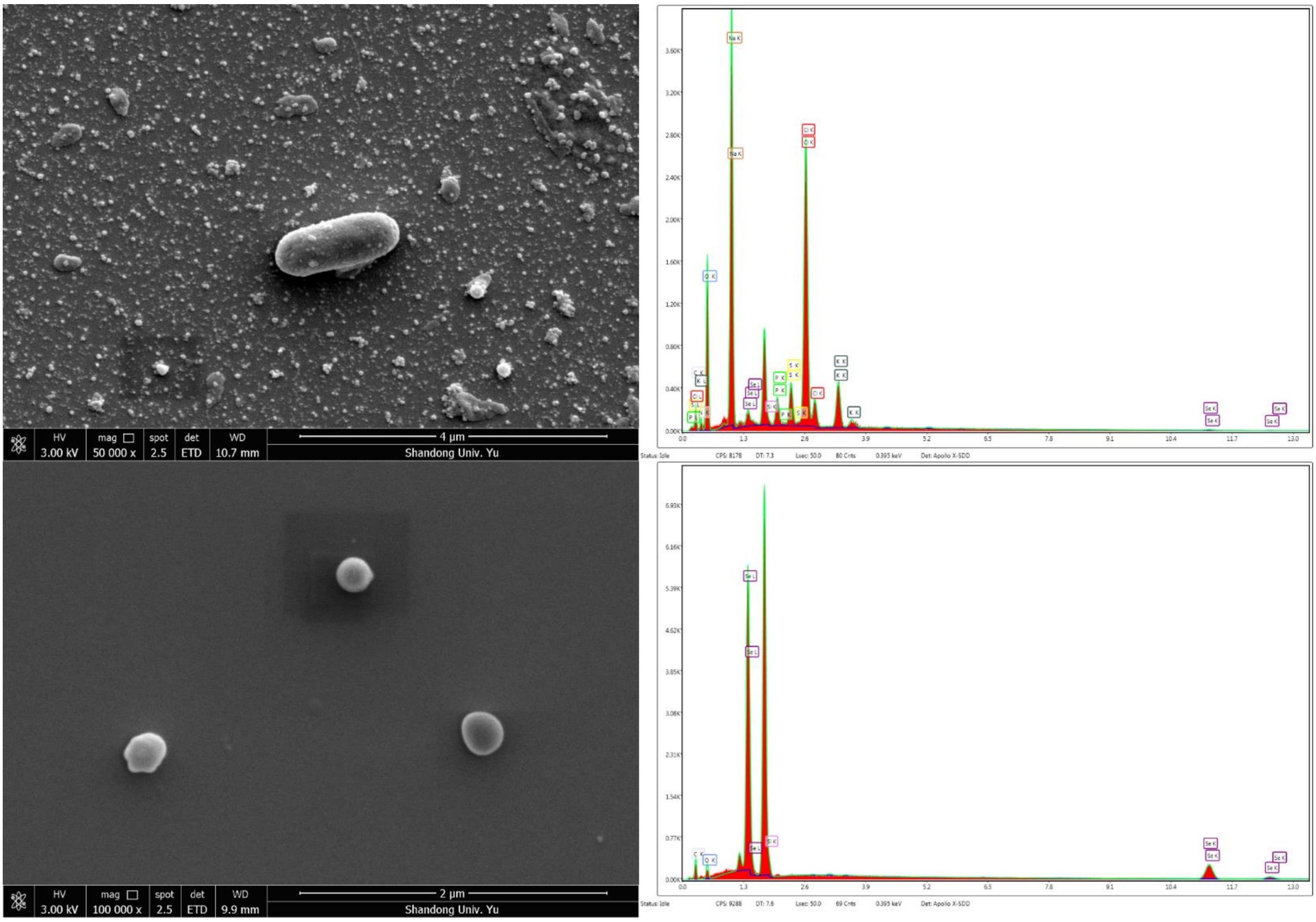

Supplementary Figure 1 SEM images and corresponding EDS spectra of bacteria culture with SeNPs (a) and the purified SeNPs (b)

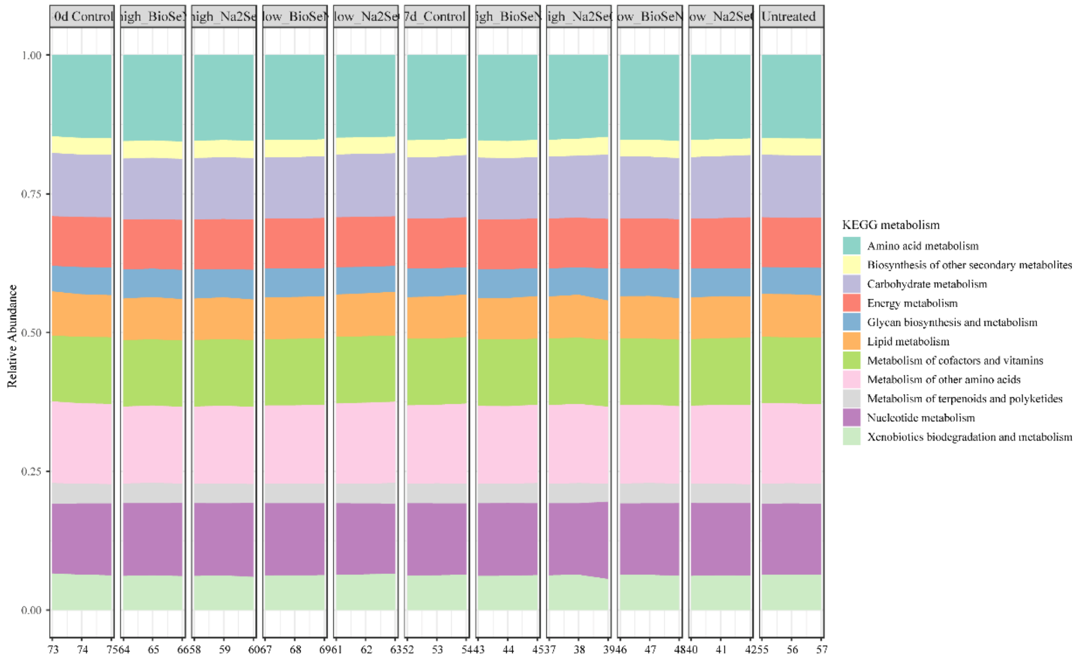

Supplementary Figure 2 Functional community composition by each experimental group

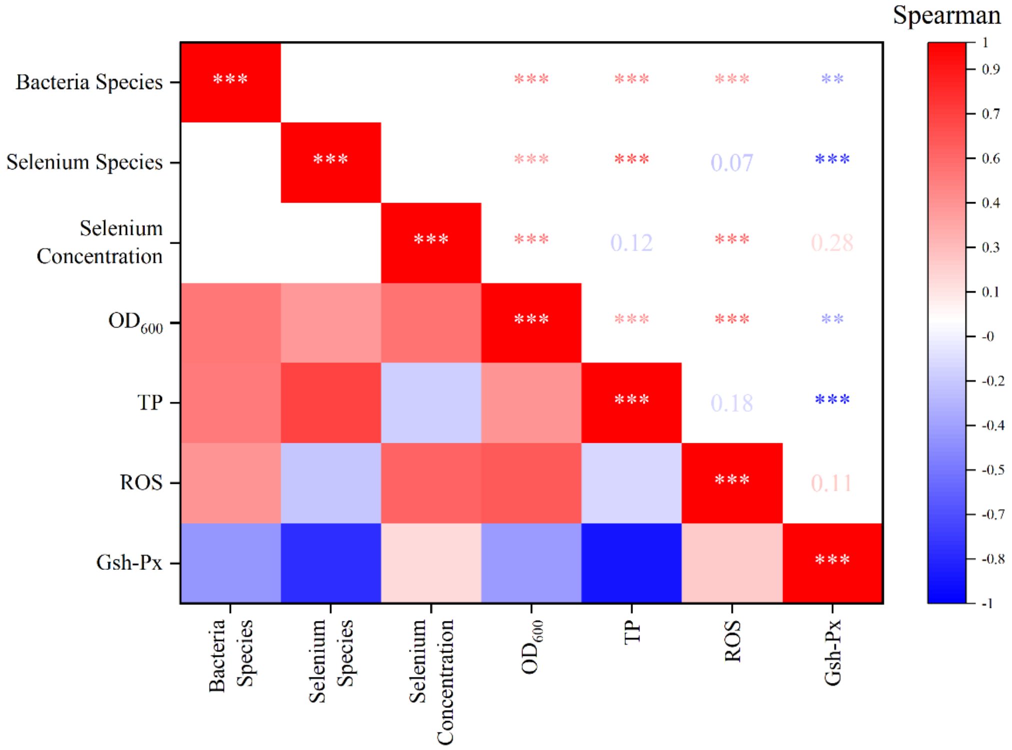

Supplementary Figure 3 Correlation analysis among bacterial species, Se species, Se concentration and Bacteria physiological indexes. *. **.***. represent significant correlation at the level of 0.05, 0.01 and 0.001, respectively.

Supplementary Table 1 Physical and chemical properties of experimental soil

Properties	Units	Value
EC	mS/m	20
pH	-	7.5
Nitrate N	mg/kg	195
Ammonia N	mg/kg	140
Available P	mg/kg	165
Total P	mg/kg	380
Available K	mg/kg	550
Total K	mg/kg	660
Available Mg	mg/kg	40
Total Mg	mg/kg	70
B	mg/kg	1.5
Mo	mg/kg	5.5
Cu	mg/kg	6
Mn	mg/kg	10.5
Zn	mg/kg	3.5
Fe	mg/kg	40

Supplementary Table 2. Primers used for PCR amplifications in this study

Name	Primer			
	Sequence of the primer	Target gene	Expected amplicon (bp)	Annealing (ºC)
K90-q	GCGGTGGAGCATGTGGTTTA	16S rRNA qPCR	181	55.0
K94-q	GATAAGGGTTGCGCTCGTTG			
515F	GTGCCAGCMGCCGCGG	16S rRNA High-throughput sequencing	392	55
907R	CCGTCAATTCMTTTRAGTTT			

Supplementary Table 3 Alpha diversity indexes of each experimental group

	97%			
	Observed OUT	Chao1 Index	Shannon Index	Simpson Index
Untreated	2177.67±115 b[1]	2846.83±115 b	5.75±0.16 b	0.99±0 b
7d Control	1916±164.04 ab	2598.17±164.04 ab	5.8±0.07 b	0.99±0 b
7d low SeNPs	1923±119.66 ab	2547.31±119.66 ab	5.71±0.06 b	0.99±0 b
7d low SeO_3^{2-}	2138.33±153.58 ab	2853.94±153.58 ab	5.67±0.36 b	0.99±0.01 b
7d high SeNPs	2025.67±75.65 ab	2797.57±75.65 ab	5.67±0.17 b	0.99±0 b
7d high SeO_3^{2-}	2181.67±146.13 b	2817.05±146.13 b	5.86±0.09 b	0.99±0 b
30d Control	1967.67±79.41 ab	2575.13±79.41 ab	5.61±0.1 b	0.99±0 b
30d low SeNPs	1964.67±399.15 ab	2439.71±399.15 ab	5.84±0.16 b	0.99±0 b
30d low SeO_3^{2-}	1644.67±127.16 a	2218.45±127.16 a	5.01±0.11 a	0.98±0 b
30d high SeNPs	2125.33±117.3 ab	2915.24±117.3 ab	5.79±0.07 b	0.99±0 b
30d high SeO_3^{2-}	1858.67±142.33 ab	2393.62±142.33 ab	4.99±0.32 a	0.95±0.02 a

1 Means ± SE within each column in each trial followed by the same letters are not significantly different (P > 0.05) according to ANOVA.

Supplementary Table 4 g-factor of EPR

	g-factor	
	Day 7	Day 30
CK	2.00416	2.00497
Low Na_2SeO_3	2.00504	2.00504
High Na_2SeO_3	2.00501	2.00501
Low SeNPs	2.00499	2.00499
High SeNPs	2.00502	2.00502
